# Retrospective Analysis of Prevalence of Tumors in Dogs and Cats in Lithuania

**DOI:** 10.3390/vetsci12111038

**Published:** 2025-10-27

**Authors:** Nomeda Juodžiukynienė

**Affiliations:** Department of Veterinary Pathobiology, Faculty of Veterinary Medicine, Veterinary Academy, Lithuanian University of Health Sciences, A. Mickevičiaus St. 9, LT-44307 Kaunas, Lithuania; nomeda.juodziukyniene@lsmu.lt

**Keywords:** tumors, mammary gland, skin, dog, cat, epidemiology, clinicopathology

## Abstract

**Simple Summary:**

Similarly to humans, the incidence of tumors in dogs and cats is steadily increasing, as companion animals are exposed to comparable environmental carcinogens. Mammary gland and skin tumors are the most frequent neoplasms in both species, with mammary carcinomas representing a major cause of mortality in dogs. A critical issue is late recognition, as early lesions are often misinterpreted by owners—or occasionally by veterinarians—as inflammatory or traumatic processes, leading to delayed veterinary care. With veterinary oncology rapidly advancing in Lithuania, early detection is essential to reduce the risk of metastasis and improve outcomes. This study evaluated the predominant tumor types and their distribution by age, sex, and species using the WHO classification system.

**Abstract:**

The objective of this study was to assess the prevalence and clinicopathological characteristics of tumors in dogs and cats in Lithuania. A total of 3525 routine diagnostic samples collected from 1 January 2020 to 1 July 2025 were microscopically examined and statistically analyzed. Tumor prevalence was slightly higher in dogs (59.2%, *n* = 1693) compared with cats (56.5%, *n* = 375). Mammary gland tumors, skin tumors, and sarcomas were the most common neoplasms in both species, although notable interspecies differences were identified. In particular, feline mammary carcinomas were more aggressive, and squamous cell carcinoma was significantly more frequent in cats. These findings are consistent with international epidemiological trends and highlight the importance of species-specific diagnostic and management strategies.

## 1. Introduction

To date, no large-scale study has evaluated the incidence of tumors in dogs and cats in Lithuania. Previous investigations have been limited to small cohorts, focusing mainly on canine skin and mammary gland tumors or feline cutaneous squamous cell carcinoma [1,2,3,4]. Accurate epidemiological assessment is hindered by several factors, including the absence of a national tumor registry, loss of data when samples are submitted abroad, and the reluctance of some owners to pursue histopathological examination due to financial constraints.

With veterinary oncology developing rapidly in the country, early tumor detection is essential to improve clinical outcomes and prevent metastasis of malignant neoplasms. The incidence and mortality of cancer are increasing in both humans and animals and are expected to continue rising [5]. Because dogs share the same environment and carcinogenic exposures as humans, they are considered valuable comparative models for drug development and clinical trials [6]. Dogs spontaneously develop mammary gland tumors (MGTs), which closely resemble human breast cancer (HBC) in their clinical and epidemiological features, making them an informative model of carcinogenesis [7,8,9,10,11]. Canine mammary tumors are particularly common, accounting for 50–70% of all neoplasms in non-spayed female dogs, primarily affecting animals over 7 years of age [12]. In cats, mammary tumors represent up to 17% of all neoplasms [13,14], with an estimated annual incidence of 13–25 cases per 100,000 cats [14]. Certain breeds, such as Siamese and Burmese, are genetically predisposed to mammary carcinomas [15,16].

Although feline physiology and biology differ from those of dogs, research on feline tumors remains valuable in comparative oncology. Human and feline carcinomas, as well as pre-neoplastic lesions, share numerous molecular mechanisms [17]. Cats also develop spontaneous tumors within a relatively short lifespan and are exposed to similar environmental carcinogens, making them useful—albeit distinct—models of carcinogenesis. Furthermore, feline mammary carcinomas are more aggressive than canine counterparts, occurring in up to 96% of cases, and cats may serve as models for HER2-targeted therapies. Felines are also particularly informative in the study of oral squamous cell carcinoma and soft tissue sarcomas of inflammatory origin, the latter being rare in dogs [18].

The spectrum of tumor types differs between species and humans: breast, lung, colorectal, and prostate carcinomas predominate in humans, whereas mammary carcinomas, mast cell tumors, and lymphomas are most frequent in dogs, and mammary carcinomas, squamous cell carcinomas, and sarcomas are most frequent in cats [19].

Intensive research is ongoing worldwide, and new diagnostic and therapeutic approaches are being developed in veterinary oncology. Several countries have already established animal cancer registries, which improve epidemiological surveillance and clinical practice [20,21,22,23,24].

The aim of this study was to evaluate the prevalence of tumors in dogs and cats in Lithuania and to analyze their distribution by breed, age, sex, and anatomical location.

## 2. Materials and Methods

### 2.1. Ethics Statement

Because the study was not based on direct contact with animals (the pathological material for routine diagnostics was used), approval from an animal ethics committee was not requested. The animals were handled by owners according to high ethical standards and national legislation (Animal Welfare Protection and Law of the Republic of Lithuania: Žin., 1997, Nr. 108-2728; 2012, Nr. 122-6126). Consent from heads of veterinary clinics and the Pathology Center was obtained to allow the use of animal tumor tissues for scientific purposes.

### 2.2. Analyzed Material

The study was based on 3525 routinely submitted diagnostic samples from dogs and cats collected from 1 January 2020 to 1 July 2025. Samples consisted of various tissues and organs submitted to the Pathology Center, Department of Veterinary Pathobiology, Veterinary Academy, Faculty of Veterinary Medicine, Lithuanian University of Health Sciences, from multiple small animal clinics. All samples were fixed in 10% neutral buffered formalin, embedded in paraffin, sectioned, and stained with hematoxylin and eosin (H&E) for histopathological examination. Information provided in the accompanying cover letters was also reviewed. Since not all accompanying documents provided detailed information about the appearance of the lesion, anatomical localization, animal breed, and castration, such statistical data were calculated from the submitted cases but not from the entire sample of one or another animal species. Due to limited data, the effect of castration on mammary gland tumors in dogs and cats has not been studied.

This study’s retrospective nature and reliance on archived pathology submissions may introduce selection bias linked to owner finances and referral patterns. As all histological grading was performed by a single pathologist, inter-observer variability was eliminated but cross-validation between multiple reviewers was not possible. Future registry-based designs should include multi-observer calibration and digital quality control to improve reproducibility.

Tumor diagnoses were formed through the use of ICD-O, the international standard for human oncology, and Vet-ICD-O, its veterinary adaptation, ensures standardized tumor coding and allows cross-comparison between human and veterinary epidemiology. These systems are increasingly used in veterinary registries to improve consistency, data comparability, and international collaboration [25,26]. For histological grading, the Elston–Ellis system (adapted) remains a well-established method for assessing mammary carcinomas, while for feline mammary tumors, a novel grading system has been recently proposed to better capture species-specific features [27,28].

The Kiupel system was used to evaluate canine cutaneous mastocytomas, which ensures greater repeatability and prognostic accuracy [29]. Because not all submission documents contained complete clinical information (e.g., lesion appearance or size), detailed statistics could not be calculated in all cases; instead, the available data were used to assess general patterns and clinical trends. In cases with diagnostic uncertainty or grading ambiguity, slides were re-evaluated jointly to reach a consensus diagnosis. Routine internal quality control was maintained to ensure grading consistency across evaluators.

The histological grade of squamous cell carcinoma in dogs and cats was established using an adapted version of Nagamine et al. [30] and Broder ‘s grading system [31] at ×100–400 magnification.

Paraffin blocks were fabricated using Shandon Pathcentre (Thermo Fisher Scientific, Waltham, MA, USA) and TES 99 (Medite Medizintechnik GmbH, Burgdorf, Germany) equipment. Serial 4-μm sections were prepared with a Sakura Accu-Cut SRM microtome (Sakura Finetek Europe B.V., Alphen aan den Rijn, The Netherlands) from each sample and used for routine H&E and Giemsa (for mast cells) staining.

### 2.3. Statistical Analysis

All statistical analyses were conducted to evaluate differences in tumor distribution across species, sex, anatomical localization, and age. Categorical variables were compared using Pearson’s chi-square test, and Fisher’s exact test was applied when any expected cell frequency was below five. Age-related comparisons between groups were performed using the non-parametric Mann–Whitney U or Kruskal–Wallis tests. For each major tumor category, the odds ratio (OR) and 95% confidence interval (CI) were calculated to estimate the relative likelihood of occurrence between species or sexes. All statistical analyses were performed using IBM SPSS Statistics, version 29.0 (IBM Corp., Armonk, NY, USA).

Statistical significance was set at *p* < 0.05, and all tests were two-tailed. Only complete histopathological cases containing sufficient numeric and categorical information were included in the analysis; incomplete or cytology-only records were excluded. Missing values were handled pairwise. All statistical computations were performed using IBM SPSS Statistics v29.0 (IBM, Chicago, IL, USA) and Python v3.11 (using the scipy.stats and statsmodels libraries). All data entries were cross-validated to ensure consistency between the histopathological reports and the statistical dataset

To improve transparency, a Supplementary Table S5 summarizing all analyzed cases has been included.

## 3. Results

A total of 3525 histopathological samples were examined, including 2861 canine and 664 feline submissions (Table 1 and Table 2). Tumors were identified in 1693 dogs (59.2%) and 375 cats (56.5%), with no statistically significant difference in overall prevalence between species (χ^2^ = 0.89, *p* = 0.32). Most submissions originated from animals aged 4–15 years, whereas samples from very young or geriatric individuals were uncommon. Non-neoplastic lesions were excluded from subsequent analyses.

Mixed-breed animals predominated in both species. In dogs, 1369 (47.8%) were mixed-breeds and 1918 (52.2%) belonged to recognized pure breeds, with a slight predominance of females. Among cats, mixed-breeds accounted for 54.8% of cases and pure breeds for 45.2%.

**Figure 1:**
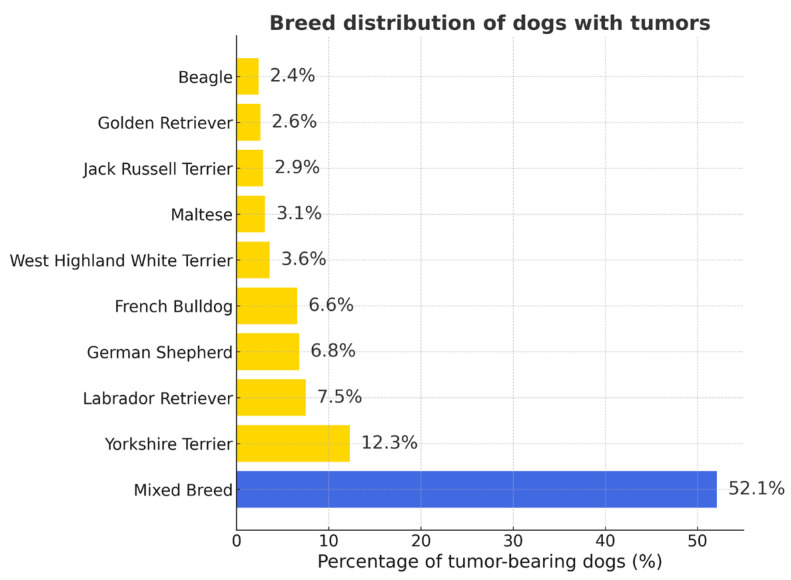
Breed distribution of dogs with tumors. Mixed-breed dogs predominated (52.1%), followed by Yorkshire Terriers, Labrador Retrievers, and German Shepherds. Percentages are given relative to all tumor-bearing dogs (*n* = 2861).

Yorkshire Terriers were the most numerous breed overall, followed by Labrador Retrievers, German Shepherds, and French Bulldog. Other common breeds included French Bulldogs, Maltese, and Jack Russell Terriers (Table S1 and Figure S1). Percentages in Figure 1 represent the proportion of each breed among tumor-bearing dogs (*n* = 2861); values do not indicate breed predisposition.

**Figure 2:**
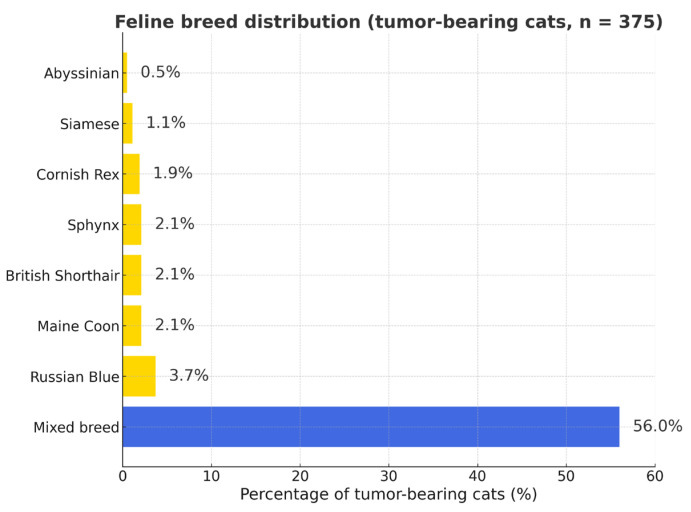
Feline breed distribution among tumor-bearing cats (*n* = 375). Percentages represent the proportion of each breed within the total number of cats diagnosed with tumors (not a measure of breed predisposition). Blue bar—mixed-breed cats; yellow bars—purebred cats.

Mixed-breed cats accounted for the majority of tumor-bearing individuals, followed by Maine Coon and British Shorthair breeds (Figure 2 and Figure S2).

Scottish Fold, Persian, Bengal, Oriental Shorthair, Peterbald, Scottish Straight, Burmese, Devon Rex, Somali, Ragdoll, Abyssinian, American Curl, Norwegian Forest Cat, and Sphynx breeds were each represented by one to seven cases (Table S2).

**Figure 3:**
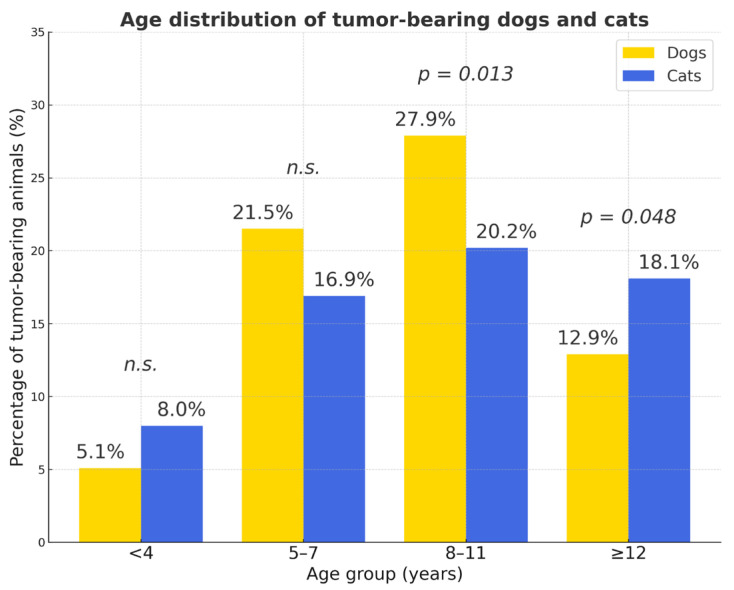
Age distribution of tumor-bearing dogs and cats. The 8–11-year age group showed the highest proportion of tumor-bearing animals in both species, whereas tumors were rare in individuals younger than 4 years. Overall, cats with tumors were significantly older than dogs (χ^2^ = 35.4, *p* < 0.001). Yellow bars—dogs; blue bars—cats; *p* values indicate statistically significant differences between species within each age group; *n.s.*—not significant.

The distribution age in dogs and cats and of tumor occurrence by age is presented in Tables S3 and S4 and in Figure 3. The majority of tumors occurred in middle-aged and older animals (4–15 years), a trend consistent across both species. Older animals (>10 years) had a significantly higher tumor risk (OR = 4.82). Tumors were infrequent in animals younger than four years and relatively rare in those older than fifteen years.

The 8–11-year age group showed the highest proportion of tumor-bearing animals in both species, whereas tumors were rare in individuals younger than 4 years. Overall, cats with tumors were significantly older than dogs (odds ratio (OR ≈ 0.32); χ^2^ = 35.4, *p* < 0.001). The proportion of cases with unrecorded sex was low in both species (<4%). Purebred dogs showed a higher risk compared to mixed-breed dogs (OR = 1.48). Mixed-breed cats had lower odds (OR ≈ 0.67)

**Figure 4:**
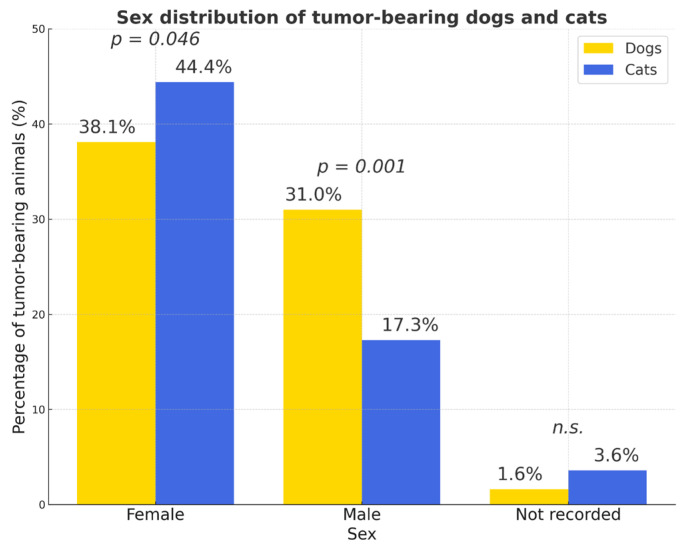
Sex distribution of tumor-bearing dogs and cats. The proportion of cases with unrecorded sex was low in both species (<4%). Percentages were calculated relative to the total number of animals within each species (dogs: *n* = 2861; cats: *n* = 664). Females represented the majority of tumor-bearing animals in both species. Yellow bars—dogs; blue bars—cats; *p* values indicate statistically significant differences between species; *n.s.*—not significant.

Females represented the majority of tumor-bearing animals, particularly in dogs (OR = 2.74) (Figure 4).

**Figure 5:**
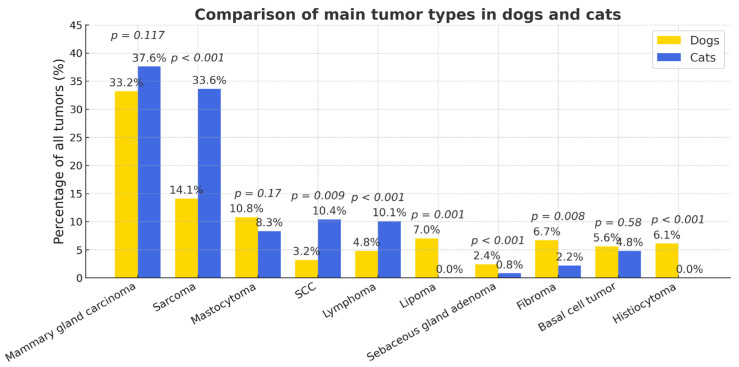
Comparative prevalence of major tumor types in dogs and cats. Percentages are calculated relative to tumor-bearing animals within each species (dogs *n* = 1693; cats *n* = 375).

Statistically significant interspecies differences were observed in the prevalence of several tumor types (Figure 5). Compared to dogs, cats had a markedly higher likelihood of developing sarcomas (OR = 3.08; 95% CI 2.39–3.97; *p* < 0.0001), squamous cell carcinoma (OR = 3.52; 95% CI 2.30–5.41; *p* < 0.0001), and lymphoma (OR = 2.24; 95% CI 1.50–3.36; *p* < 0.001). Conversely, lipomas (OR = 0.33; 95% CI 0.16–0.65; *p* = 0.001), sebaceous gland adenomas (OR = 0.11; 95% CI 0.04–0.36; *p* < 0.001), fibromas (OR = 0.37; 95% CI 0.18–0.76; *p* = 0.008), and histiocytomas (OR = 0.02; 95% CI 0.001–0.33; *p* < 0.001) were significantly more frequent in dogs. Population-level prevalence (% of all examined animals) showed a similar ranking of tumor types, though with proportionally lower absolute values (Figure S3). The risk of malignancy increased with age (OR = 1.56). The relative risk of skin and subcutaneous tumors (RR = 1.85) was higher for dogs.

When considering only females, the proportion of mammary carcinomas was slightly lower in dogs (37.12%) than in cats (41.23%) (*p* > 0.05; χ^2^ = 0.078, RR = 1.22, 95% CI 0.97–1.54; not significant). The most frequently affected dog breeds were Yorkshire Terriers, German Shepherds, Maltese Bichons, and crossbreeds. When both dogs and cats were grouped by age, a clear trend was observed indicating that mammary tumors were more frequent in older cats (χ^2^(4) = 35.43, *p* = 3.8 × 10^−7^; Cramér’s V = 0.224; OR/RR + 95% CI). The distribution of mammary carcinoma by age group in dogs was as follows: 1–5 years—8.9%, 6–8 years—23.7%, 9–11 years—38.1%, 12–14 years—25.6%, and 15–20 years—3.7%. In cats, the respective proportions were: 1–5 years—9.9%, 6–8 years—19.9%, 9–11 years—24.8%, 12–14 years—29.1%, and 15–20 years—16.3%. These data show a tendency toward a higher frequency of mammary tumors in older cats, although the smaller number of geriatric animals may partly reflect lifespan limitations.

Mammary gland lesions exhibited marked species-specific differences in distribution and biological behavior. Benign lesions were significantly more common in cats (21.28%) than in dogs (5.16%) (*p* < 0.001; OR = 4.91, 95% CI [2.34–10.32]). Malignant lesions predominated in both species but showed a higher degree of aggressiveness in cats, where simple carcinoma accounted for 77.3% of all mammary gland pathologies compared with 56.6% in dogs (*p* = 0.002; OR = 2.57, 95% CI [1.42–4.67]). *Lymphangiosis carcinomatosa* was proportionally more frequent in cats (25.5%) than in dogs (4.5%), indicating a greater metastatic potential in feline tumors (*p* < 0.001; OR = 7.4, 95% CI [3.1–17.8]). In dogs, the histological spectrum was more heterogeneous, including complex and mixed carcinomas with bone or cartilage metaplasia, whereas in cats, simple carcinomas clearly predominated. These findings confirm the well-documented tendency for feline mammary tumors to present as uniformly malignant and invasive lesions, contrasting with the broader morphological variability seen in canine cases.

**Figure 6:**
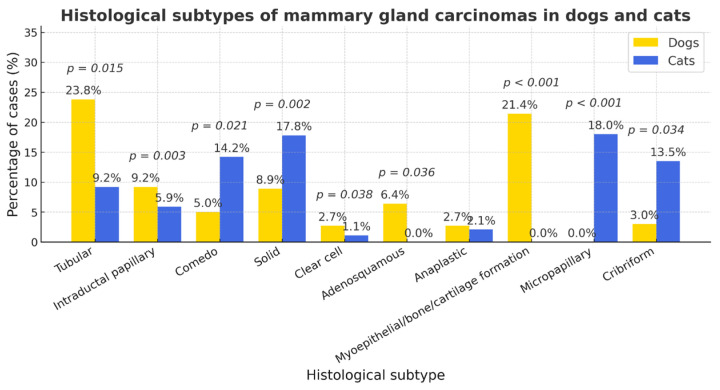
The distribution of histological subtypes of mammary carcinomas in dogs and cats. Cats exhibited a significantly higher proportion of comedo, solid, cribriform, and micropapillary carcinomas (*p* < 0.01), whereas dogs showed greater prevalence of tubular, papillary, myoepithelial, and osseocartilaginous variants (*p* < 0.01). Clear-cell type was also more frequent in cats (*p* = 0.041). Other differences were not significant (*n.s.*).

When comparing histological subtypes of mammary gland carcinomas marked interspecies differences were observed(Figure 6). Tubular carcinoma was more frequent in dogs (17.6%, 95% CI 14.5–20.7) than in cats (9.2%, 95% CI 4.3–14.1; *p* = 0.028; OR ≈ 2.1). In contrast, comedo (cats 14.2%, 95% CI 8.4–20.0 vs. dogs 5.0%, 95% CI 3.1–6.9; *p* = 0.001; OR ≈ 0.32) and solid (cats 17.8%, 95% CI 11.2–24.4 vs. dogs 8.9%, 95% CI 6.6–11.2; *p* = 0.009; OR ≈ 0.46) patterns were significantly more common in cats. Cribriform (cats 13.5% vs. dogs 2.7%; *p* < 0.001; OR ≈ 0.18) and micropapillary (cats 10.6% vs. dogs 0%; *p* < 0.001; Fisher) variants also predominated in cats. Conversely, myoepithelial/benign-part components (dogs 21.4% vs. cats 0%; *p* < 0.001; Fisher), bone/cartilage formation (dogs 8.9% vs. cats 0%; *p* < 0.001; Fisher), and intraductal papillary type (dogs 5.9% vs. cats 0%; *p* = 0.004; Fisher/χ^2^) occurred essentially only in dogs. Clear cell morphology was more frequent in cats (6.4% vs. 2.7%; *p* = 0.041; OR ≈ 0.41), whereas anaplastic and adenosquamous types showed no reliable between-species difference at available sample sizes (both *n.s.*). Overall, cats exhibited a shift toward more aggressive/poorly differentiated architectures, while dogs showed greater morphological diversity with frequent mixed or complex features.

**Figure 7:**
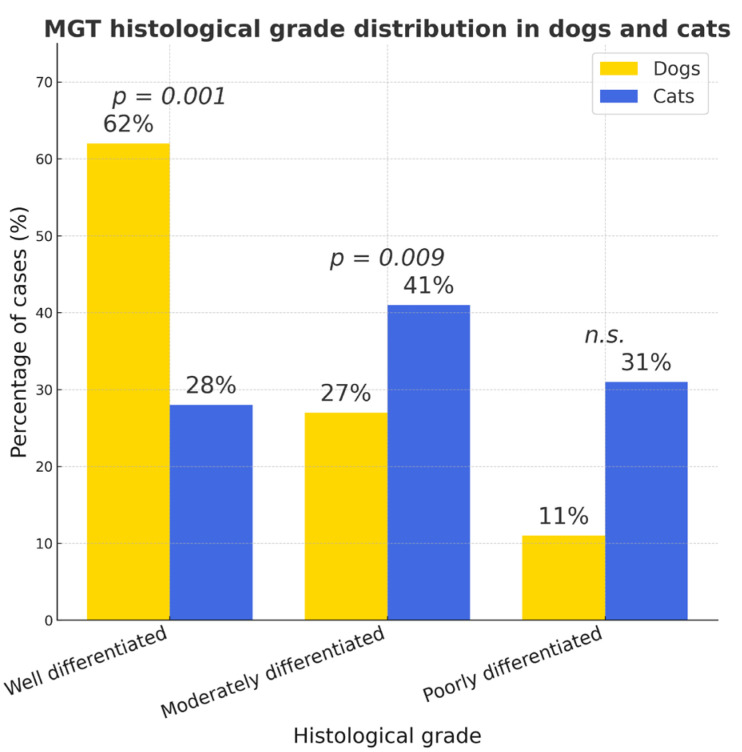
MGT (mammary gland tumor) histological grade distribution in dogs and cats. Between-species differences were significant for well- (*p* < 0.001) and moderately differentiated tumors (*p* = 0.009) but not for poorly differentiated carcinomas (*n.s.*).

Well-differentiated mammary gland carcinomas were significantly more prevalent in dogs (62%, 95% CI 57.7–66.1%) than in cats (28%, 95% CI 19.7–36.3%; *p* < 0.001), whereas cats showed higher proportions of moderately (41%) and poorly (31%) differentiated tumors (*p* = 0.009, *n.s.*, respectively) (Figure 7).

**Figure 8:**
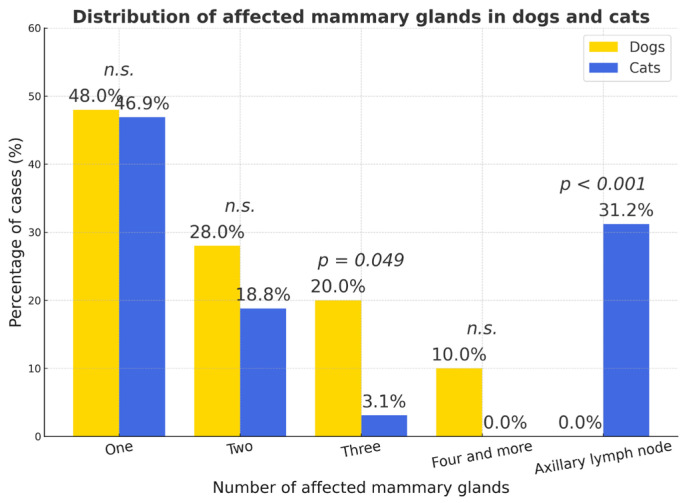
Distribution of affected mammary gland number in dogs and cats. Percentages represent the proportion of cases within each species; *p*-values indicate statistical significance (*n.s.*—not significant).

Because not all case records included detailed information on tumor size or the number of affected glands, statistical calculations and graphs were based on the available subset of data. Data on the number of mammary glands affected were available for 250 dogs and 64 cats. Tumor size measurements were available for 202 dogs and 62 cats. Most tumors in both species involved a single mammary gland (dogs—48.0%, 95% CI = 41.8–54.2; cats—46.9%, 95% CI = 34.5–59.6). Multifocal involvement of two or more glands was more frequent in dogs (two glands—28.0%, 95% CI = 22.5–33.5; three—20.0%, 95% CI = 15.1–24.9) than in cats (two—18.8%, 95% CI = 9.6–28.0; three—3.1%, 95% CI = 0–7.3; *p* < 0.05). Axillary lymph-node involvement occurred exclusively in cats (31.2%, 95% CI = 19.7–42.7; *p* < 0.001) (Figure 8).

**Figure 9:**
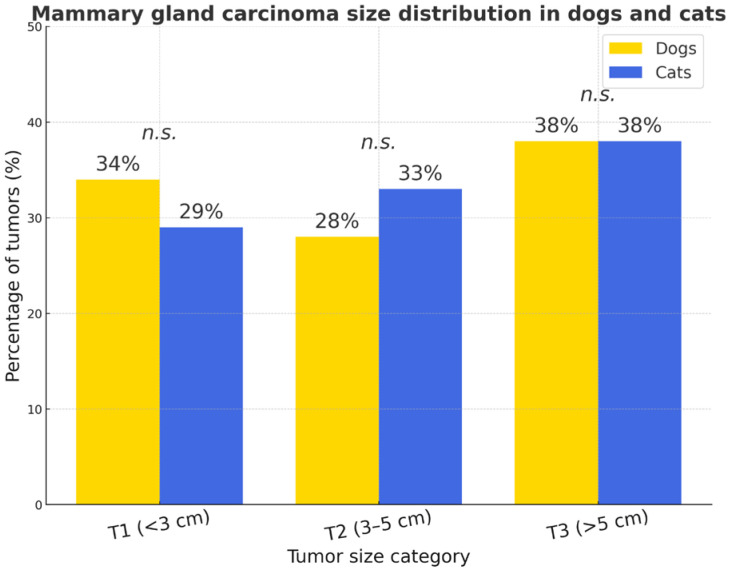
Mammary gland carcinoma size distribution in dogs and cats. No statistically significant interspecies differences were detected (*p* > 0.05, *n.s.*).

According to the size distribution, no significant species difference was observed. Large tumors (T3, >5 cm) predominated in both species, T2 lesions were slightly more prevalent in cats, and T1 tumors were more common in dogs. The 95% confidence intervals for tumor size proportions overlapped across all categories (T1–T3), indicating no significant interspecies difference (*p* > 0.05). The widest uncertainty range was observed in cats due to smaller sample size (*n* = 62) (Figure 9).

Due to limited data, the effect of neuter status on MGT occurrence could not be evaluated. The findings indicate a higher overall malignancy grade in feline mammary tumors compared to canine cases.

**Figure 10:**
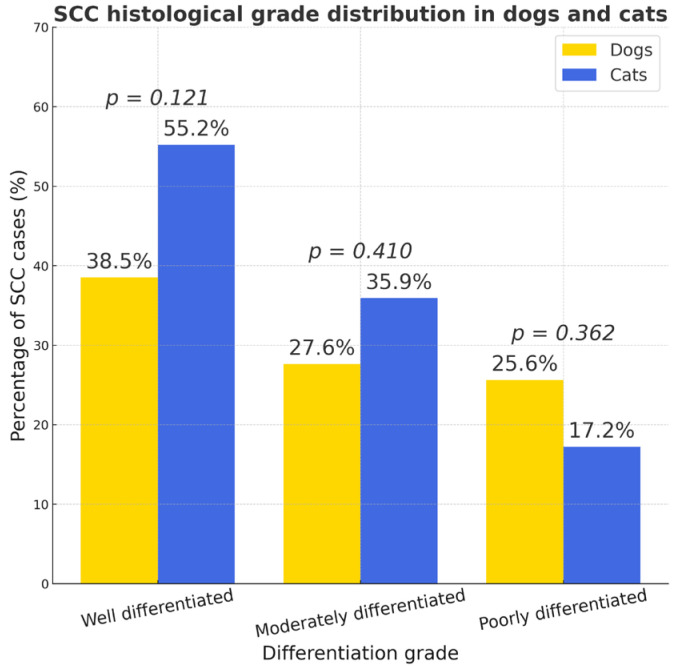
Histological grade distribution of squamous cell carcinoma (SCC) in dogs (*n* = 58) and cats (*n* = 39). Bars show the proportion of well-, moderately-, and poorly differentiated SCC within each species, with exact percentages annotated.

SCC occurred significantly more often in cats (χ^2^ = 7.05, OR = 2.72, 95% CI 0.31–0.82; *p* < 0.01) (Figure 5): in dogs (*n* = 58), SCC accounted for 3.19% of all tumors (2.03% of the total number of dogs), while in cats (*n* = 39) it represented 10.4% of all feline tumors (5.87% of all cats). The histological grading of SCC was evaluated using an adapted version of the systems proposed by Nagamine et al. [29] and Broder [30], under ×100–400 magnification. In dogs, well-differentiated carcinomas predominated, whereas in cats both well- and moderately differentiated types were common. However, there was no statistically significant difference in grade distribution between species: ninety-five percent confidence intervals (CI) were: dogs—well 26.0–51.0%, moderate 23.5–48.3%, poor 14.3–36.9%; cats—well 39.6–70.8%, moderate 13.6–41.6%, poor 5.3–29.1%. No statistically significant interspecies differences were detected for any grade (two-proportion tests: well *p* = 0.121; moderate *p* = 0.410; poor *p* = 0.362). The overall 3 × 2 association was non-significant (χ^2^ ≈ 2.44, *p* ≈ 0.295) (Figure 10).

In dogs, squamous cell carcinomas (SCC) were most frequently located on the gums, neck, and tail, whereas in cats they predominantly affected oral sites (gums, tongue, and palate), as well as the ear and sinuses. Females were slightly more affected than males in both species, but the differences were not statistically significant (dogs: *p* = 0.19, 95% CI −8.4–39.6%; cats: *p* = 0.84, 95% CI −25.8–30.1%). The overall distribution of tumor sites differed significantly between species (χ^2^ = 14.87, *p* = 0.036), reflecting species-specific anatomical predilections. SCCs in cats showed a clear tendency to develop in the oral cavity, while in dogs they were more commonly found on the body surface and extremities. The reliability of these comparisons is limited by the small sample sizes within several anatomical subgroups. When comparing the influence of age, a statistically significant difference was observed, indicating that cats with SCC were slightly older than dogs (Mann–Whitney U test: *p* = 0.016; Cliff’s δ = 0.30; 95% CI [0–4 years]). No statistically significant association was found between sex and age in either species. In dogs, age did not differ between females and males (*p* > 0.05). Among cats, females tended to be approximately two years older than males, although the difference was not statistically significant (OR = 1.04, 95% CI 0.64–1.70; *p* = 1.000).

In dogs, basal cell tumors (the benign variant now classified as trichoblastoma, while the malignant form remains basal cell carcinoma) (*n* = 68) accounted for 2.38% of all examined animals and 4.02% of all canine tumors. In cats, basal cell tumors (*n* = 17) represented 2.56% of all animals and 4.76% of all feline tumors. A significantly higher proportion of basal cell carcinomas was observed in cats (64.7%, *n* = 11) compared to dogs (36.8%, *n* = 25). Although the proportion of carcinomas among basal cell tumors in cats was nearly twice that in dogs, the difference reached only marginal statistical significance (*p* ≈ 0.054). The overall incidence of basal cell tumors was similar between species (OR = 0.58, 95% CI 0.36–0.94; *p* = 0.029). In dogs, basal cell tumors and carcinomas occurred equally in both sexes, most often between 6 and 9 years of age. In cats, these tumors were typically diagnosed in older individuals (~9–11 years), with a slight predominance in females. Mixed-breed animals were most frequently affected, although cases also occurred in small-breed dogs (Yorkshire Terriers, Bichons) and in purebred cats (Siamese, Bengal, Persian). Histologically, the following patterns were identified: in cats—solid, cystic, heavily pigmented, ribbon, and medusoid types; in dogs—predominantly ribbon (*n* = 12) and medusoid (*n* = 10) variants. Statistical comparisons of subtype frequencies were not applicable due to small sample sizes.

Pilomatrixomas were identified in 1.51% of all dogs (*n* = 43), representing 2.45% of all canine tumors. In cats, only one case was recorded (0.15% of all cats; 0.28% of all feline tumors). Pilomatrixomas were significantly more common in dogs than in cats (all animals: OR = 10.26, 95% CI 1.41–74.8; *p* = 0.0047; among tumor-bearing animals: OR = 9.23, 95% CI 1.28–66.4; *p* = 0.0074). These findings confirm that pilomatrixomas represent a rare tumor type in both species, with a clear predominance in dogs, particularly in females (*n* = 24).

Sebaceous gland tumors were significantly more frequent in dogs (3.88%, *n* = 111) compared with cats (0.45%, *n* = 3) among all examined animals (OR = 9.37, 95% CI 2.87–30.5; *p* < 0.001). They accounted for 6.65% of all canine tumors and 0.84% of all feline tumors. Among the sebaceous gland tumors in dogs, Meibomian gland tumors represented 10.81% (*n* = 12). Female dogs comprised 55.34% (*n* = 63), males 39.80% (*n* = 48), and 6.80% (*n* = 7) were of unrecorded sex. Adenomas predominated (61.4%, *n* = 70), followed by epitheliomas (20.18%, *n* = 23) and carcinomas (18.42%, *n* = 21).

Hepatoid gland tumors (*n* = 87) were diagnosed in 3.04% of all dogs and accounted for 5.14% of all canine tumors. A clear male predominance was observed (82.76% males, 16.1% females, and 1.5% of unknown sex) (OR = 5.73, 95% CI 2.81–11.7; *p* < 0.001). Adenomas were the most common (56.3%, *n* = 49), followed by epitheliomas (27.6%, *n* = 24) and carcinomas (16.1%, *n* = 14). A statistically significant age difference was found among adenomas, epitheliomas, and carcinomas (Kruskal–Wallis test: H = 8.34, *p* = 0.015), indicating that older dogs were more likely to develop carcinomas. Sex had no significant effect on tumor type (χ^2^ = 5.88, *p* = 0.208).

Anal sac modified apocrine gland adenocarcinoma was diagnosed in 11 dogs (0.39% of all dogs; 0.65% of dogs with tumors). A marked female predisposition was observed (*n* = 9). Affected dogs were 7–12 years old, mostly mixed breeds (*n* = 9). Regional lymph node metastases were frequent, involving the medial iliac, internal iliac, and sacral nodes.

Mast cell tumors (MCTs) were diagnosed in 183 dogs (6.4% of all dogs; 10.8% of canine tumors) and 31 cats (4.7% of all cats; 8.3% of feline tumors). Although proportionally more common in dogs, the interspecies difference was not statistically significant (RR = 1.30; χ^2^ = 1.85; *p* = 0.174; 95% CI 0.91–1.88). In both species, MCTs showed nearly equal sex distribution. In dogs, females accounted for 51.4% (*n* = 94) and males for 48.6% (*n* = 89) (χ^2^ = 0.14; *p* = 0.71). In cats, females comprised 51.6% (*n* = 16) and males 48.4% (*n* = 15) (χ^2^ = 0.03; *p* = 0.86; OR = 1.48; 95% CI 0.92–2.38). No significant sex-related differences were observed (*p* > 0.05). Breed analysis revealed that most affected dogs were mixed-breed (*n* = 52), followed by Labrador and Golden Retrievers (*n* = 40), French Bulldogs (*n* = 20), and Pugs (*n* = 15), which aligns with previously reported predisposed breeds in European and North American studies. In cats, crossbreeds predominated (*n* = 19), with sporadic cases in Cornish Rex (*n* = 5), Maine Coon (*n* = 3), and single cases in Bengal, Russian Blue, British Shorthair, and Sphynx cats.

In both species, MCTs were most frequently diagnosed in middle-aged animals (8–11 years; dogs: 50.8%, cats: 51.6%), while juvenile animals (<4 years) were rarely affected. No marked differences were observed between species across age groups, suggesting a comparable age-related pattern of MCT occurrence in dogs and cats.

**Figure 11:**
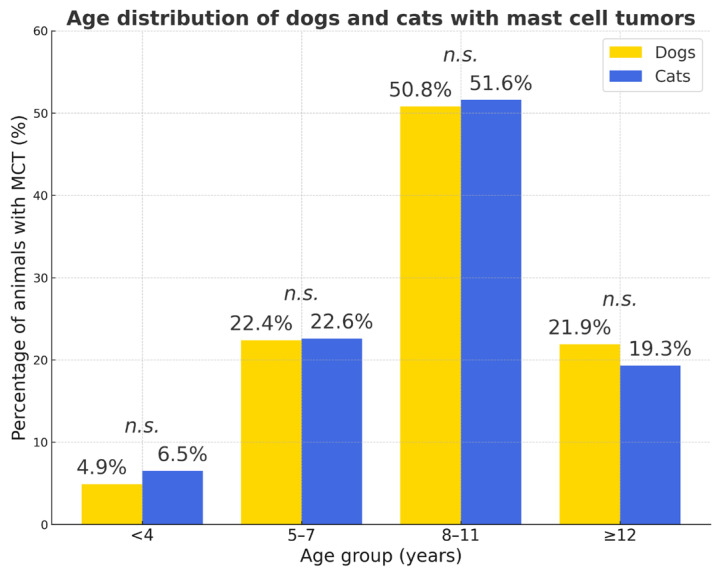
Age distribution of dogs and cats with mast cell tumors. Percentages represent the proportion of animals with MCT within each species. Statistical comparison between species showed no significant differences in any age group (*n.s.*—not significant).

The age distribution of dogs and cats with mast cell tumors is shown in Figure 11.

Dogs tended to develop MCTs slightly later than cats, but this difference was not statistically significant (Mann–Whitney U = 3038.5; *p* ≈ 0.051; OR = 1.48; 95% CI 0.92–2.38). The mean, median, and standard deviation did not differ between male and female dogs, suggesting no sex effect on age distribution. In cats, MCTs were more often detected in older females and middle-aged males, but these differences were not significant (*p* > 0.1). In dogs, cutaneous MCTs predominated, comprising 39.34% Grade I, 39.67% Grade II, and 18.03% subcutaneous forms.

The majority of MCTs were located within the dermis (skin, I grade—39.3%; skin, II grade—31.7%), while subcutaneous forms accounted for 18% of cases. Only a small proportion of tumors were recorded as unclear (6%) or complicated (4.9%) in localization.

**Figure 12:**
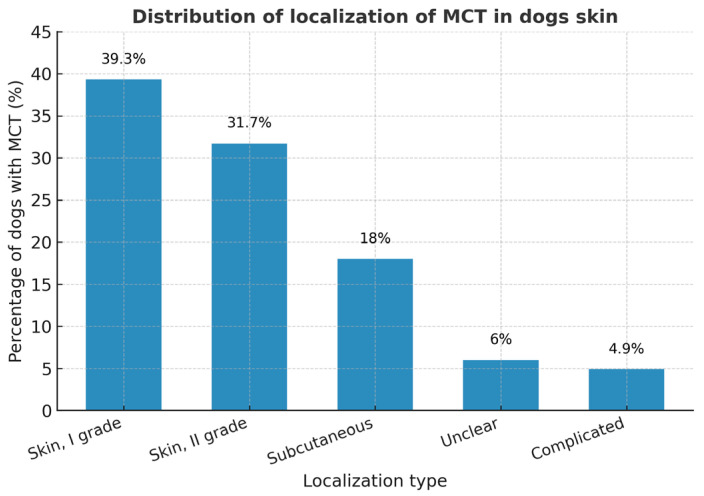
The figure shows the percentage distribution of canine cutaneous mast cell tumors (MCTs) by localization and grade. Data are presented as percentages of all canine MCT cases.

Two additional histopathological categories were identified (see Figure 12). The first, labeled “unclear”, included 6.01% of cases in which only the tumor tissue was submitted without surrounding dermal or subcutaneous structures, making precise classification impossible. The second category, termed “complicated” (4.91%), encompassed highly inflammatory and necrotic mastocytomas showing both dermal and subcutaneous infiltration. These proportions are comparable to previous European reports, where low- to intermediate-grade MCTs predominate.

In cats, cutaneous MCTs were most often located on the head, neck, and ears (*n* = 12), followed by the tail and abdomen (*n* = 10), and dorsolateral trunk (*n* = 6). Three cases lacked recorded site information. Most feline cases were consistent with the cutaneous type, which is typically less aggressive than visceral or splenic forms reported in other studies.

Lymphomas were significantly more frequent in cats than in dogs (*p* = 0.008), being approximately twice as common in relative risk terms (χ^2^ = 7.05; *p* = 0.0079; OR = 0.50; 95% CI 0.31–0.82). in dogs, 81 cases were diagnosed (2.83% of all dogs; 4.78% of canine tumors), while in cats, 36 cases were identified (5.42% of all cats; 10.08% of feline tumors). Most canine lymphomas were multicentric (95%), typically involving peripheral lymph nodes, whereas alimentary lymphoma predominated in cats (79%), followed by nasal and renal forms. There was no significant association with sex in either species. No significant age difference was found between species, although dogs were slightly older on average (Mann–Whitney U = 727.5; *p* = 0.445).

Plasmacytomas were rare, occurring in 1.58% of dogs and 0.3% of cats (one case). In dogs, 50% of cases were located in the spleen, 45% in the skin or mucous membranes, and the remaining cases in bones or internal organs. Although proportionally more frequent in dogs, the difference between species was not statistically significant (*p* > 0.05).

Histiocytomas were diagnosed in 104 dogs (3.64% of all dogs; 6.14% of canine tumors). They occurred mainly in young animals aged 6–18 months, rarely beyond 8 years. Morphologically, the tumors were typically hairless, round, and light-colored, about pea-sized, although larger ulcerated and bleeding lesions (3–4 cm) were also observed, particularly on the digits or flanks. In approximately 10% of cases, rapid growth was noted, and highly mitotically active tumors (up to 65 mitoses per 2.37 mm^2^) were observed despite dense lymphocytic infiltration. In five cases, darkly pigmented, irregularly surfaced lesions were clinically misdiagnosed as melanomas. Histiocytomas occurred across multiple breeds, with a slightly higher prevalence in mixed-breed dogs, French Bulldogs, and Labrador Retrievers. Sex distribution was 54.8% males, 42.3% females, and 2.9% unknown, indicating a slight male predominance (*p* < 0.05).

Within each species, sarcomas accounted for 14.12% and 33.61% of all tumors in dogs and cats, respectively (OR = 0.57, 95% CI 0.42–0.74; *p* < 0.001) (Figure 5).

**Figure 13:**
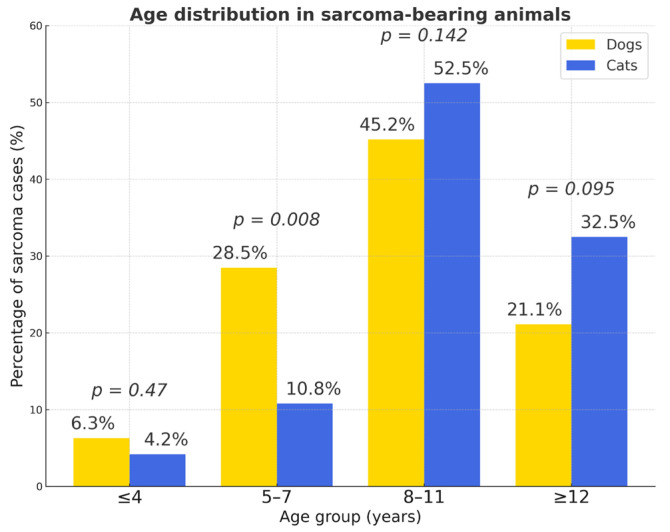
Age distribution in sarcoma-bearing animals. Percentages represent the proportion of sarcoma cases within each species; *p*-values indicate between-species comparisons for each age group (*n.s.* when *p* ≥ 0.05).

Older animals were more commonly affected. Among cats, 85.5% were aged ≥ 8 years, while only 15% (*n* = 39) were ≤7 years (χ^2^ = 13.9; *p* = 0.00018; 95% CI 1.37–4.47). Similarly, 66.3% (*n* = 156) of affected dogs were ≥8 years, confirming age as a major risk factor (χ^2^ ≈ 60.6; *p* < 0.001; OR = 4.54; 95% CI 3.09–6.67) (Figure 13). A significantly higher proportion of sarcomas occurred in dogs aged 5–7 years (*p* = 0.008), while other age groups showed no statistically significant differences (*p* > 0.05). The oldest cats (≥12 years) were more frequently affected than dogs, although this difference did not reach statistical significance.

Sex distribution was balanced in both species (dogs: χ^2^ ≈ 0.31; *p* = 0.515; OR = 0.89; 95% CI 0.61–1.28; cats: χ^2^ = 1.17; *p* = 0.56; OR = 0.86; 95% CI 0.40–1.86). Purebred dogs were significantly more often affected than mixed breeds (χ^2^ ≈ 50.9; *p* < 0.001; OR = 0.25; 95% CI 0.17–0.37).

**Figure 14:**
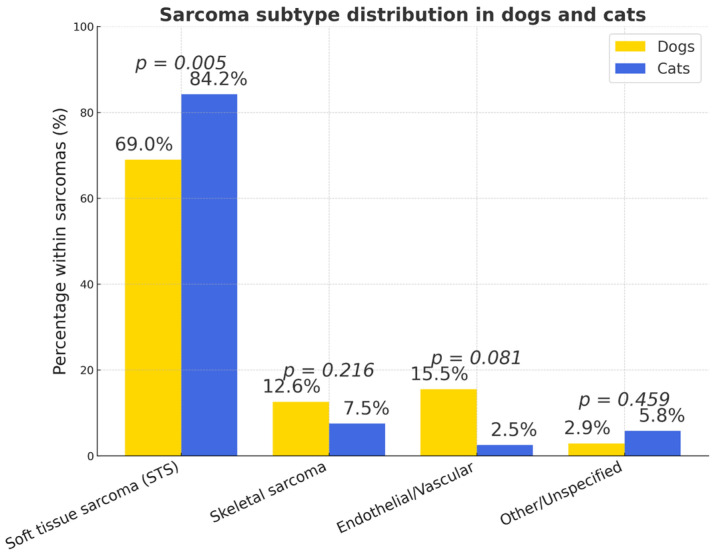
Types of sarcomas in dogs and cats (% of all subtypes). The figure presents the proportional distribution of sarcoma types in dogs and cats.

Soft tissue sarcomas represented the majority of sarcomas in both species (dogs = 69.0%, *n* = 165; cats = 84.2%, *p* < 0.005) (Figure 14). The overall prevalence of sarcomas was significantly higher in cats (32.3%, 121/375) than in dogs (14.1%, 239/1693) (*p* < 0.0001). Cats were nearly three times more likely to develop sarcomas compared to dogs (OR = 2.89; 95% CI: 2.20–3.80).

In dogs, the main histotypes of STS (soft tissue sarcoma) were perivascular wall tumors (PNST/PWT; 41.1%, *n* = 51), fibrosarcomas (27.4%, *n* = 34), and liposarcomas (20.2%, *n* = 25).

In cats, feline injection-site sarcomas (FISS) were predominant (86.1%, *n* = 87; *p* < 0.005), followed by myxosarcomas (5.9%, *n* = 6), liposarcomas (3.0%, *n* = 3), and peripheral nerve sheath or unclassified sarcomas (2.0% each, *n* = 2).

Mixed-breed cats predominated, though purebreds such as Russian Blue were occasionally affected in FISS cases. No sex predisposition was observed in FISS (*p* > 0.051). The most frequent location was the interscapular/shoulder region (57.5%, *n* = 50). Less common sites included the chest, neck, and jaw, while isolated cases occurred in the head, limbs, waist, and groin. In 11.5% (*n* = 10) of cases, localization was unspecified. Mixed-breed cats were most frequently affected, with no sex predisposition. Skeletal sarcomas accounted for 7.5% of feline cases, mainly osteosarcomas, while vascular sarcomas were rare (2.5%).

PWT most commonly affected the elbow, thigh, and paw regions and increased in frequency after 7 years of age. Most affected dogs were purebreds, particularly Labrador Retrievers, Cocker Spaniels, and German Shepherds. PWT incidence increased after 7 years of age. Females were more frequently affected (56.9%, *n* = 29) than males (39.2%, *n* = 20), though this was not statistically significant (χ^2^ = 1.33, *p* = 0.249).

The frequency of well-differentiated STS sarcomas in dogs (fibrosarcomas, liposarcomas, myxosarcomas) and cats (FISS, liposarcomas, myxosarcomas) was similar (χ^2^ ≈ 0.65, *p* > 0.3). Cats showed a slightly higher proportion of moderately differentiated sarcomas (χ^2^ ≈ 2.98, *p* = 0.053), whereas poorly differentiated sarcomas were somewhat more frequent in dogs (χ^2^ ≈ 2.86, *p* = 0.081). None of these differences reached statistical significance.

In dogs, osteosarcomas accounted for 0.8% (*n* = 23) of all animals and 1.4% of canine tumors, representing a markedly higher incidence than chondrosarcomas (0.1%, *n* = 4; 0.2% of canine tumors).

Hemangiosarcomas were identified in 37 dogs (2.2% of canine tumors and 15.15% of all dogs sarcomas) and two cats (0.6% of feline tumors and 2.5% of feline sarcomas). The spleen was the most common site in dogs (*n* = 34), while a single feline case occurred in the ventral abdominal wall.

Cutaneous hemangiomas were also observed (32 dogs, 2 cats), with no significant interspecies difference (*p* > 0.5).

A total of 127 lipomas were recorded: 118 in dogs and 9 in cats. These accounted for 4.44% of all dogs and 7.03% of all canine tumors, and 1.36% of all cats and 2.4% of feline tumors, respectively. The interspecies difference was statistically significant (*p* < 0.01): lipomas were much more frequent in dogs (χ^2^ = 12.84; *p* = 0.00034; RR = 3.27, 95% CI 1.67–6.40). Dogs had a ~3.3-fold higher risk of developing lipomas compared to cats. Although cats were slightly older at diagnosis (mean 10 years) than dogs (mean 8.8 years), the difference was not significant (t = −1.27; *p* = 0.235; Mann–Whitney U = 386.5; *p* = 0.245). In dogs, lipomas were clearly associated with middle-to-older age (6–12 years) and were more frequent in females (57%) (χ^2^ = 3.81; *p* = 0.051; Fisher’s exact test: *p* = 0.051; OR = 5.28, 95% CI 2.01–13.9; *p* < 0.001). In cats, sex distribution was almost equal (*p* = 1.0). Common localizations in cats included the side, back, and abdomen (3 cases each). In dogs, lipomas were most often found on the abdomen (*n* = 10), flanks/chest/axillary region (*n* = 38), neck (*n* = 5), and scapular area (*n* = 4). Mixed breeds (*n* = 24), Retrievers (*n* = 13), and Bulldogs (*n* = 3) were the most common breeds affected. Almost all cats were mixed breeds (8/9).

Fibromas were less frequent in cats than in dogs, comprising 3.29% (*n* = 96) of all dogs and 5.55% of canine tumors, compared to 1.20% (*n* = 8) of all cats and 2.24% of feline tumors. Most fibromas in dogs occurred in females (57.7%, *n* = 38) compared to males (39.2%, *n* = 38), with sex unrecorded in 3.1% of animals. Fibromas were distributed almost equally among purebred (49.5%, *n* = 48) and mixed-breed (45.4%, *n* = 44) dogs. Because a large proportion of animals were of unknown age (65%), age distribution should be interpreted with caution. Young dogs (≤8 years) accounted for 13.4% (*n* = 13), while older dogs (≥8 years) represented 21.6% (*n* = 21). About half of all fibromas were odontogenic (52.1%, *n* = 49).

Melanomas were detected in 1.64% of all dogs, representing 2.78% of canine tumors, and in 1.6% of all cats, representing 0.9% of feline tumors. The interspecies differences were not statistically significant (*p* > 0.87 and *p* > 0.1).

Tumors of neuroendocrine origin were diagnosed in two dogs. A single Merkel cell tumor was identified in one dog, although immunohistochemical confirmation could not be performed. Another case represented a pheochromocytoma. Both cases were incidental findings and too few for meaningful statistical evaluation. A single case of canine adrenal gland adenolipoma with extramedullary hematopoiesis was diagnosed. The lession was incidental and considered benign. Pancreatic islet carcinoma was found in one dog. Thyroid tumors were rare, comprising 3.5% of all feline tumors and 0.83% of all canine tumors. Although proportionally higher in cats, the difference was not statistically significant due to the limited number of cases. In cats, benign follicular adenomas were the prevailing type, while in dogs, malignant follicular and medullary carcinomas predominated. Testicular tumors (*n* = 20) comprised 0.7% of all dogs and 1.18% of all canine tumors. Leydig cell tumors were the most common (40%, *n* = 12), followed by Sertoli cell tumors (33%, *n* = 10), and seminomas (27%, *n* = 8). The incidence of these three types was not significantly different (χ^2^ = 0.80; *p* = 0.67), indicating a roughly even distribution without dominance of a particular type. No testicular tumors were identified in cats in this dataset.

Mesotheliomas were diagnosed in five dogs (0.17% of all dogs and 0.30% of all canine tumors). These cases represented 3.01% of all mesenchymal tumors in dogs. All cases occurred in older animals.

The incidence of colorectal carcinoma differed slightly between species. In dogs (*n* = 10), it accounted for 0.35% of all dogs and 0.59% of all canine tumors, whereas in cats (*n* = 1), it represented 0.15% and 0.28%, respectively (OR = 2.12; *p* = 0.701). Although dogs showed a higher number of cases, the difference was not statistically significant due to the small sample size. In both species, histological examination revealed carcinoma arising from pre-existing mucosal polyps, suggesting a progression from benign rectal mucosal polyp to adenocarcinoma. Among the nine canine cases, three were French Bulldogs, and rectal polyps were also detected in five dogs.

Overall, the comparative statistical analysis demonstrated significant species-related differences in several tumor categories. Lymphomas and squamous cell carcinomas were more frequently diagnosed in cats, whereas mast cell tumors, lipomas, and mammary gland carcinomas predominated in dogs. These findings reflect both species-specific biological predispositions and diagnostic submission trends. No significant differences were found for melanomas, fibrosarcomas, and hemangiomas, while some tumor categories showed limited sample size preventing robust statistical interpretation.

## 4. Discussion

This study represents the first large-scale analysis of tumor prevalence and clinicopathological patterns in dogs and cats in Lithuania. Despite the absence of a national veterinary cancer registry, incomplete documentation in submitted cases, and the frequent use of cytology instead of histopathology, the dataset provides valuable insight into species-, age-, and tumor-type-specific trends that are largely consistent with data from other European and America regions [5,19,32,33]. Although multiple statistical tests (χ^2^, Mann–Whitney U, Fisher’s exact, OR/RR ± CI) were applied for interspecies, sex, and age group comparisons, certain small subgroups limited statistical power. Thus, some trends should be interpreted cautiously.

As this investigation was based on retrospective submissions to a single academic pathology center, the material may not fully represent the national population or the distribution of referral versus primary-care cases. Submission bias related to owner finances, regional accessibility, and clinician sampling practices cannot be excluded. Such biases are inherent in retrospective histopathology-based studies and should be considered when extrapolating prevalence estimates to the general pet population.

In cats, mammary gland carcinoma and sarcoma predominated, whereas in dogs, mammary gland carcinoma and mastocytoma were most frequent. The odds of developing malignancy were approximately 1.5 times higher in older animals. Female dogs had higher odds of developing tumors (OR = 2.74). Purebred dogs had about 1.5 times higher odds (OR = 1.48) compared to mixed-breed dogs. Dogs accounted for a slightly higher proportion of tumors than cats, consistent with their higher population share in clinical practice. However, cats showed a higher relative cancer risk when tumor prevalence was calculated among examined individuals, as has also been observed elsewhere [19,33,34,35]. Lower diagnostic submission rates in cats likely reflect species-specific behavioral and owner-related factors, including stress during transport and lower inclination toward advanced diagnostics [19,33,35].

Mammary gland carcinomas were the most frequent neoplasms in both species, accounting for approximately one-third of all tumors, similar to reports from Europe and South America [36,37,38,39,40,41].

In dogs, simple and complex carcinomas predominated, whereas cats exhibited a higher proportion of high-grade, solid, and comedo-type tumors with lymphangiosis carcinomatosa, confirming their more aggressive biological behavior. Feline cases occurred in older animals (mean: 10.7 years) than canine ones (mean: 9.6 years), in agreement with age-associated risk documented in previous studies [12,39,42].

Clinically, canine mammary carcinomas display wide histologic heterogeneity—ranging from benign mixed to invasive simple carcinomas—necessitating grading and staging for accurate prognosis. Feline mammary carcinoma, conversely, is almost uniformly malignant, and metastases or multiple gland involvement are common at diagnosis. The predominance of single-gland involvement in cats but multifocality in dogs in this study underscores biological and hormonal distinctions between the species. Early ovariohysterectomy remains the most effective preventive measure [5,12,39,42].

SCC was significantly more frequent in cats than in dogs, particularly involving auricular, oral, and sinonasal sites, whereas in dogs, it predominated in gingiva and digit or tail skin. These patterns parallel published data highlighting the role of ultraviolet (UV) exposure in unpigmented feline skin and trauma-related pathogenesis in canine digits [43,44,45,46,47,48].

UK and US cohorts confirm that feline SCC is enriched at UV-exposed sites (pinnae, nasal planum) and tends to present later and with higher histological grade than canine SCC—paralleling our older median age and site distribution in cats. Digit and gingival SCC in dogs are likewise highlighted in UK series, often linked to trauma and pigment-related risk, supporting our species-specific topography [49].

Male cats were more frequently affected, likely reflecting behavioral factors such as outdoor exposure and territorial activity.

Histologically, feline SCCs were more often moderately or poorly differentiated, while dogs showed a predominance of well-differentiated lesions. Cats with SCC were, on average, two years older, reinforcing the association between cumulative UV exposure, chronic inflammation, and malignant transformation.

Clinically, feline oral and auricular SCCs are often diagnosed late, when curative surgery is no longer feasible, underscoring the need for early recognition of chronic non-healing oral or cutaneous lesions. In dogs, gingival SCC must be differentiated from chronic periodontal or granulomatous lesions, as inflammatory keratin granulomas may mimic neoplastic proliferation. Veterinarians should be alert if the animal has a skin formation that periodically ruptures, similar to an abscess. In particular, veterinarians should educate cat owners about non-healing wounds, especially in the ear/muzzle area.

MCT ranked among the most frequent cutaneous neoplasms in both species, comprising approximately 10–11% of canine and 8% of feline tumors, consistent with data from other European countries [50,51,52,53]. No significant sex predisposition was detected. Breed distribution corresponded to known genetic risks—Labrador and Golden Retrievers, Pugs, and French Bulldogs—supporting the role of KIT pathway mutations in predisposed breeds. Mixed-breed prevalence in this dataset mirrors the general population structure in Lithuania. Age distribution was typical: most cases occurred in middle-aged dogs (7–10 years), while cats showed a slightly younger onset but broader range. Histologically, low- and intermediate-grade cutaneous forms predominated, whereas subcutaneous MCTs were less frequent, probably underrepresented due to incomplete excision or cytology-only submissions. The “unclear” and “complicated” histological categories, characterized by diffuse dermal infiltration and necrosis, highlight diagnostic limitations in fragmented samples. Feline cutaneous MCTs were mainly located on the head, neck, and trunk, aligning with previously described non-visceral forms, while splenic and intestinal variants were rare [50].

Lymphomas were significantly more frequent in cats than in dogs, with a twofold relative risk, corroborating findings from other European studies [52,53,54,55,56,57,58,59,60].

Feline cases were mainly alimentary or extranodal (nasal, renal), whereas canine lymphomas were predominantly multicentric. These patterns align with the well-known species distinction—alimentary, FeLV/FIV-associated forms in cats versus B-cell multicentric forms in dogs. No sex or age predisposition was evident, though cats tended to present slightly younger. Clinically, early cytological and immunophenotypic testing remains crucial to avoid underdiagnosis of alimentary and extranodal lymphoma in cats.

Histiocytomas were observed exclusively in dogs, primarily in young animals (6–18 months), confirming their benign, self-limiting nature. They were most frequent in mixed-breed, French Bulldog, and Labrador populations, with a mild male predominance, echoing reports suggesting hormonal or genetic influences.

Despite their benign course, larger ulcerated or pigmented histiocytomas were occasionally misdiagnosed as melanomas, emphasizing the role of histopathology for definitive diagnosis [23,60,61].

Sarcomas were approximately twice as common in cats as in dogs (33.6% vs. 14.1% of all tumors), largely reflecting the high prevalence of feline injection-site sarcomas (FISS). In cats, soft tissue sarcomas predominated (84%), and the interscapular/shoulder region was the most frequent localization—consistent with the well-established post-vaccinal pathogenesis of FISS [62,63,64,65,66,67,68,69]. Older cats were significantly more affected (≥8 years), and no sex predisposition was observed. These findings align with international recommendations promoting distal-limb or tail injection sites to facilitate radical excision if sarcomas occur [70].

In dogs, soft tissue sarcomas were the main group (69%), including perivascular wall tumors (PWTs), fibrosarcomas, and liposarcomas. PWTs predominated (41%) and were most often located on the limbs, thorax, and trunk, frequently in middle-aged and older animals. Unlike cats, dogs also exhibited a noticeable proportion of endothelial sarcomas (mainly hemangiosarcomas) and skeletal sarcomas (osteosarcomas, chondrosarcomas). These patterns correspond to reports from other European registries [5,20,21,22,23,24].

Although the grade distribution was similar between species, cats showed a slightly higher proportion of moderately differentiated sarcomas, while dogs had more poorly differentiated lesions—suggesting species-specific biological variability in tumor aggressiveness.

Vascular sarcomas (hemangiosarcomas) were rare in cats but more frequent in dogs, particularly in the spleen and subcutis. The presence of both benign (hemangiomas) and malignant vascular tumors in dogs underlines the need for histological differentiation in epidemiological datasets.

Basal cell and trichoblastic tumors were common in both species, but the malignant variant (basal cell carcinoma) was almost twice as frequent in cats. This emphasizes the need for histological confirmation even in apparently benign dermal nodules. Sebaceous and hepatoid gland tumors were confined to dogs, with the expected male predominance in hepatoid lesions due to androgen dependence [21,23,71,72].

Anal sac apocrine gland adenocarcinomas were rare but clinically relevant, occurring mainly in females with nodal metastases, a pattern previously described in European cohorts [23]. Regional nodal metastasis was observed (medial iliac, internal iliac, and sacral lymph nodes). The incidence of this tumor differed from that reported in the literature—about 2% of all skin tumors and 17% of tumors in the perianal region [72].

These findings indicate a clear species-related predilection for mesenchymal and epithelial tumor types in cats and for benign mesenchymal and histiocytic neoplasms in dogs. The overall tumor spectrum and prevalence patterns in Lithuania closely resemble those reported in Europe, indicating that the distribution of companion animal cancers is relatively consistent across regions when adjusted for population structure and diagnostic access. From a clinical standpoint, the results highlight the need for early biopsy or cytological examination of any persistent lesion, regardless of presumed etiology. For cats in particular, owner education on early detection of oral, auricular, and injection-site lesions could substantially improve outcomes.

Several limitations must be acknowledged. The data were derived exclusively from histopathological submissions, introducing sampling bias toward surgically treated or excised lesions and underrepresenting tumors diagnosed solely through cytology. Case documentation was often incomplete, limiting analysis of recurrence, metastasis, or treatment outcomes. Breed representation reflects the local pet population rather than standardized epidemiological sampling. Additionally, the absence of a national veterinary tumor registry in Lithuania restricts direct comparison with population-based incidence data.

Despite these limitations, the present dataset provides the first structured overview of tumor types and histopathological characteristics in Lithuanian companion animals, aligning with European prevalence patterns and offering a baseline for future national cancer registry development.

## 5. Conclusions

This study provides the first large-scale overview of tumor prevalence in dogs and cats in Lithuania. Mammary gland carcinomas, squamous cell carcinomas, mast cell tumors, lymphomas, and sarcomas were the most common neoplasms, with distinct species-specific patterns. Canine mammary tumors showed greater histological diversity, while feline mammary carcinomas were predominantly high-grade and more aggressive. Squamous cell carcinoma was significantly more frequent in cats, lymphomas were mostly multicentric in dogs but alimentary in cats, and sarcomas were particularly common in cats due to injection-site tumors.

These findings highlight the importance of early detection, standardized histological grading, and awareness of species-specific differences to improve treatment outcomes. They also emphasize the need for a national veterinary tumor registry and the integration of molecular prognostic markers to strengthen clinical practice and comparative oncology research within the “One Health” framework.

Future perspectives should focus on the integration of Lithuanian data into multicenter veterinary oncology studies, which would allow more robust cross-country comparisons, identification of breed- and region-specific risk factors, and the development of harmonized preventive strategies.

## Figures and Tables

**Table 1:** Dog breeds and gender distribution.

	Male	Female	Sex Unknown (X)
Mixed breed	354	492	15
Pure breed	811	977	65
Breed unknown (X)	32	45	11

**Table 2:** Cat breeds and gender distribution.

	Male	Female	Sex Unknown (X)
Mixed breed	167	217	6
Pure breed	121	117	13
Breed unknown (X)	5	12	6

## Data Availability

The data presented in this study are available on reasonable request from the corresponding author. The data are not publicly available due to privacy restrictions related to the diagnostic archive.

## Supplementary Materials

The following supporting information can be downloaded at https://www.mdpi.com/article/10.3390/vetsci12111038/s1. Table S1: Dogs—Breed distribution (% of total). Figure S1: Most common dogs breeds in analyzed material. Table S2: Cats—Breed distribution (% of total). Figure S2: Most common feline breeds in analyzed material. Table S3: Dogs—age distribution. Table S4: Cats– age distribution. Figure S3: Prevalence of tumor types in dog and cat populations. Table S5: Full Date of Diagnosis and Animals.
