# Supplementary material for: Retrospective Analysis of Prevalence of Tumors in Dogs and Cats in Lithuania
Source: Vet Sci. 2025 Oct 27;12(11):1038. doi: 10.3390/vetsci12111038 (PMC12656924; doi:10.3390/vetsci12111038)

Supplementary Table S1. Dogs – Breed distribution (% of total)

Breed	Number of dogs	% of total dogs
Jorkšyro Terjeras	230	8.0
Labradoro Retriveris	129	4.5
Vokiečių Aviganis	125	4.4
Vakarų Škotijos Baltasis Terjeras	125	4.4
Prancūzų Buldogas	113	3.9
Maltos Bišonas	62	2.2
Džeko Raselo Terjeras	44	1.5
Vengrų Trumpaplaukis Vižlas	41	1.4
Zvergšnauceris	41	1.4
Biglis	41	1.4

Supplementary Figure S1. Most common dogs breeds in analysed material

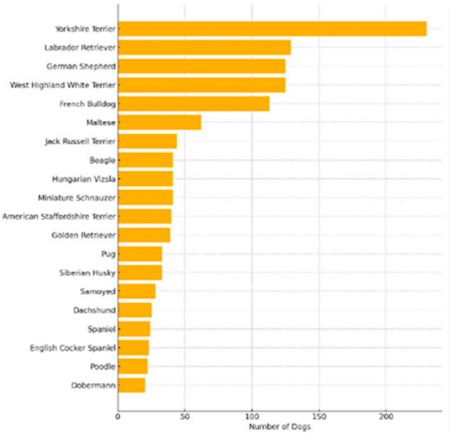

Supplementary Table S2. Cats – Breed distribution (% of total)

Breed	Number of cats	% of total cats
Meino Meškėnas	37	5.6
Britų Trumpaplaukiai	30	4.5
Rusų Mėlynoji	26	3.9
Sfinksas	23	3.5
Kornvalio Reksas	13	2.0
Abisinijos	11	1.7
Siamo Katė	10	1.5
Škotų Nulėpausė	7	1.1
Bengalijos	6	0.9
Persų Katė	5	0.8

Supplementary Figure S2. Most common feline breeds in analysed material

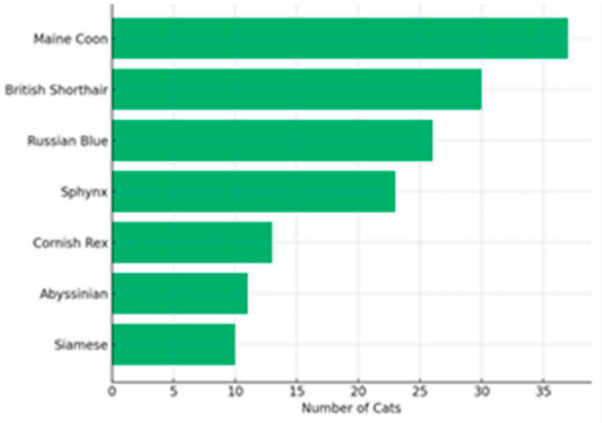

Supplementary Table S3. Dogs – age distribution

Group of age	Number of dogs
<1 y.	62
1–3 y.	153
4–7 y.	875
8–10 y.	871
11–15 y.	759
>15 y.	35
Age unknown	106

Supplementary Table S4. Cats– age distribution

Group of age	Number of cats
<1 y.	30
1–3 y.	64
4–7 y.	187
8–10 y.	146
11–15 y.	210
>15 y.	32
Age unknown	69

Supplementary Figure S3. Prevalence of tumor types in dogs and cats populations.

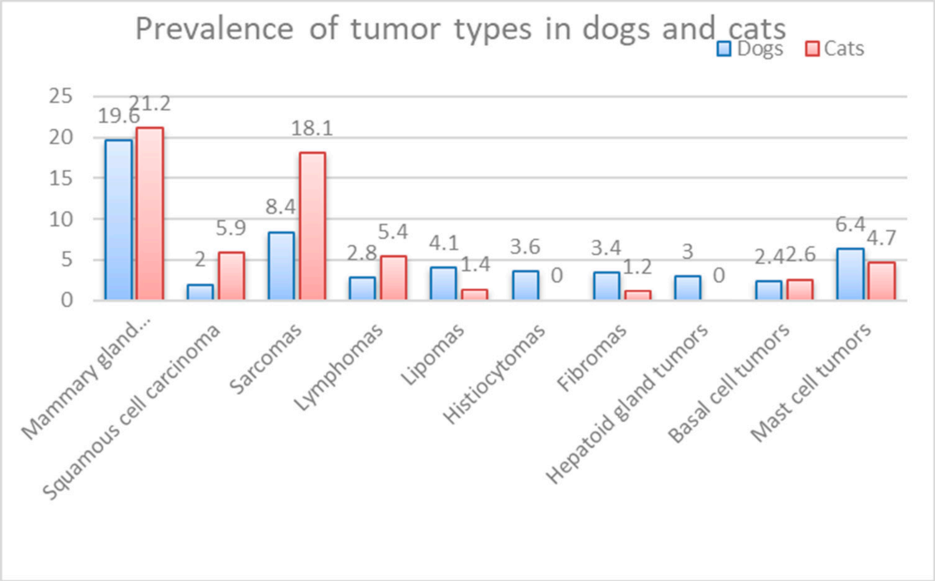

Supplement: Supplementary file 1 [file vetsci-12-01038-s001.zip › vetsci-3899997-SupplementaryMaterial.pdf]
